# Enterprise negotiation and communication management system under the guidance of the Internet of Things

**DOI:** 10.1371/journal.pone.0284891

**Published:** 2023-04-25

**Authors:** Huijun Chen

**Affiliations:** Wuhan City Polytechnic, Wuhan City, China; Universiti Teknologi Malaysia, MALAYSIA

## Abstract

The multi-agent system is used to study the negotiation problem of virtual enterprises in the context of the Internet of Things (IoT) to strengthen the decision-making ability of enterprises and improve the negotiation efficiency between different enterprises. Firstly, virtual enterprises and high-tech virtual enterprises are introduced. Secondly, the virtual enterprise negotiation model is implemented using the agent technology in the IoT, including constructing the operation mode of the alliance enterprise agent and the member enterprise agent. Finally, a negotiation algorithm based on improved Bayesian theory is proposed. It is applied to virtual enterprise negotiation, and the effect of the negotiation algorithm is verified by setting an example. The results show that: (1) When one side of the enterprise adopts a risk-taking strategy, the number of negotiation rounds between the two sides increases. (2) High joint utility can be achieved when both parties to the negotiation adopt a conservative strategy. (3) The improved Bayesian algorithm can improve the negotiation efficiency of enterprises by reducing the number of negotiation rounds. This study aims to achieve efficient negotiation between the alliance and the member enterprises to improve the decision-making ability of the alliance owner enterprise.

## Introduction

The rapid development trend of economic globalization and the intensification of market internationalization make the economic environment more and more complex. These require enterprises not only to adapt to the market changes but also to carry out more flexible restructuring and resource allocation in the scope of the global market. Thanks to the swift growth of information technology, a new cross-organization management mode, High-tech Virtual Enterprise (HTVE), which combines enterprise core competence with computer network technology, is growing and developing rapidly. However, in the whole construction and operation process of HTVE, on the one hand, due to the incompleteness of inter-enterprise and intra-enterprise infrastructure, the communication between enterprises is not convenient. On the other hand, due to the imperfection of information, task coordination, and unequal distribution of interests, there are many cooperation conflicts in the operation of the HTVE alliance. These conflicts must be solved in time to ensure that the smooth operation of the alliance is maintained. Therefore, it is urgent to establish a set of perfect Negotiation Support System (NSS) to alleviate many contradictions brought about by market changes.

Currently, the most researched virtual enterprise is the choice of its partners [1]. The research is generally divided into two parts. The first is the evaluation indicator system selected by virtual enterprise partners. In this part, scholars have different perspectives, so the proposed evaluation indicators are also various [2]. For example, scholars have established an evaluation system based on the core competency principles selected by partners, the principles of summarizing cost accounting, agility, and risk minimization [3]. The second is the comprehensive evaluation method of virtual enterprise partners. Commonly used evaluation methods include the Analytic Hierarchy Process (AHP), fuzzy comprehensive evaluation, fuzzy optimization, neural network, timing multi-objective decision-making method, trigonometric analysis-based AHP, packet network method, integer programming, and Genetic Algorithm (GA) [4]. However, these evaluation methods are based on a single-entity enterprise. Experts have researched how to apply them to virtual enterprises formed by multiple entities. Initially, a dynamic alliance process model is constructed [5]. A dynamic alliance is a temporary alliance formed by several essentially independent enterprises to achieve a common goal. There is a common interest between the alliance members until the common goal is accomplished, after which the alliance will be dissolved. Based on this model, the virtual enterprise organization design model is proposed [6]. On account of analyzing and weighing the advantages and problem forms of virtual enterprise, the model uses Integration Definition for Function 3 (IDEF3) methods to conduct general modeling and process description for the organizational design process of virtual enterprise. Subsequently, a dynamic reconstruction model of virtual enterprises based on multiple agents is designed. This model realizes the dynamic management of virtual enterprise on the basis of Agent theory and achieves good results [7].

The NSS was proposed as a concept and attracted the attention of many scholars in the late 1970s [8]. Since the advent of NSS, there has yet to be a commonly recognized, systematic, and precise definition. Nonetheless, it is widely believed that the NSS as a computer-based system can improve the efficiency and effectiveness of negotiations [9]. Later, the NSS was considered a human-computer interaction system. It mainly applies decision-making theory, behavioral science, information technology, ergonomics, and other technical theories and methods to study the negotiation situation and strategically prepare negotiators before negotiations [10].

To sum up, although the current research on the use of the multi-objective GA in the decision-making mechanism of the alliance enterprise has made some progress, it still only stays in the stage of simple theoretical knowledge and has not implemented the model. Therefore, based on previous studies, this study uses Agent technology and the Bayesian algorithm to implement a relevant model, thereby solving the negotiation and decision-making problems during the establishment of virtual enterprises, and sets an example to verify the proposed model. The innovation is to improve the Bayesian self-learning negotiation algorithm in the negotiation algorithm and successfully apply it to the virtual enterprise multi-topic negotiation process. The purpose of this study is to provide a theoretical basis for the further improvement of the decision-making ability and negotiation effect of enterprises.

## Literature review

Virtual enterprise exists as a complex and open system, and its research focus is on structural analysis and process modeling. Ouyang (2021) analyzed the relevant factors in the whole process of virtual enterprise construction [11]. Le et al. (2021) proposed the optimal utilization method of resources during the operation of virtual enterprises. At the same time, they pointed out some crucial factors of constructing a virtual enterprise, such as partner strategy; partner selection, external relations, organizational structure among partners, and related supporting policies [12]. On this basis, Vila et al. (2021) proposed key factors in the construction process of virtual enterprises, including market opportunity identification, core competence identification, strategic partner selection, enterprise structure reconstruction, agility measurement, organizational operation mode, etc. [13]. Gan and Yin (2021) believed that the constructed virtual enterprise model is a kind of abstract information language to describe agile enterprise cooperation, while the current research focuses on the modeling process focuses on its method and content [14].

According to the current research status, there are more achievements in partner selection of virtual enterprises, mainly because the evaluation indexes and methods involved in partner selection are more and more abundant. Additionally, there are many research results on the core competitiveness of virtual enterprises. However, most of them focus on the explanation of principles. There needs to be more research on identifying, developing, cultivating, and managing competitiveness, and a lack of practical application on virtual enterprises’ process analysis and construction. It is especially pointed out that there are few achievements in the research of production planning and task allocation in virtual enterprises. Most scholars adopt Agent technology-based to realize virtual enterprises’ production planning and task allocation in the existing literature. But the actual practice still imitates the traditional enterprise’s task allocation and plan-making without highlighting the characteristics of virtual enterprises themselves.

## Methods

### High-Tech Virtual Enterprise (HTVE)

To conduct the follow-up research better, this study first introduces the theoretical basis needed, including the concept of virtual enterprise and HTVE.

(1) Virtual enterprise

A virtual enterprise is a temporary alliance initiated by an alliance and several independent business partners for a market opportunity. Each partner contributes to its core areas, such as design, manufacturing, and distribution, and realizes cost-sharing and sharing of cooperation results [15]. A virtual enterprise is not an independent legal entity but a dynamic and temporary alliance of multiple independent enterprises established. The organization is about to disintegrate when the desired goals are achieved [16].

(2) HTVE

HTVE is a dynamic consortium of multiple member enterprises driven by interests, which is a networked organization [17]. In the operation process, the member enterprises are based on cooperative relations due to the independence of each member enterprise. It is more likely that the behavior of member companies will deviate from the strategy of the entire HTVE due to changes and interference of environmental factors. Therefore, HTVE has a great deal of instability. It is also easy to form various conflicts between member enterprises [18]. The temporary, dynamic, and intercultural nature of the HTVE complicates the conflict issues of HTVE members [19]. HTVE involves exchanging and sharing resources and capabilities between multiple enterprises from conception, formation, and operation to dissolution. In this form of cooperation, there may be inconsistencies between the alliance partners in managing objectives and tasks, the process of virtual management, the processing of knowledge, and the distribution of benefits. Thereby, conflicts will inevitably arise [20].

### MAS-based virtual enterprise negotiation communication model

After introducing the relevant theories of virtual enterprise technology, this section is based on this theory and combines multi-agent technology to construct the enterprise negotiation and communication model. It involves model construction, the structure construction of the Agent in a virtual enterprise Multi-Agent System (MAS) model, communication between agents in a virtual enterprise model, and other modules design.

(1) MAS

1) The concept of the MAS

MAS was born out of the book Administrative Behavior. The book mentions that a large organization organizes many to make up for the limited ability of a single agent to work. Similarly, the division of labor and the fact that each agent is responsible for a dedicated task can compensate for the limited ability of a single agent to learn new tasks. The organized flow of information between social institutions can compensate for the limited capacity of individual agents to process information and use it to communicate. Although the theory is aimed at human society, it lays the ideological foundation for the MAS system. The intelligence of a single agent is limited, but the agents can be organized through the appropriate architecture to make up for the shortcomings of each agent. Then, the entire system’s capabilities exceed any single agent’s capabilities [21].

There are two keys to a MAS system. One is the collaboration between agents, and the other is the adaptation to the environment (information environment). Establishing a protocol set for exchanging information between agents to work together is necessary. Agent collaboration typically comes in the task and result sharing [22].

2) Architecture of the MAS

The architecture of the agent describes the following. The first is the essential components of the agent and its role. The second is the mechanism of connection and interaction of each component. The third is determining the different actions the agent should take through the perceived internal state and external environment. The fourth is the effect of the agent’s behavior on its internal form and external environment [23]. The currently proposed agent architecture can be roughly divided into two categories: inferential architecture and reactive architecture.

**Fig 1:**
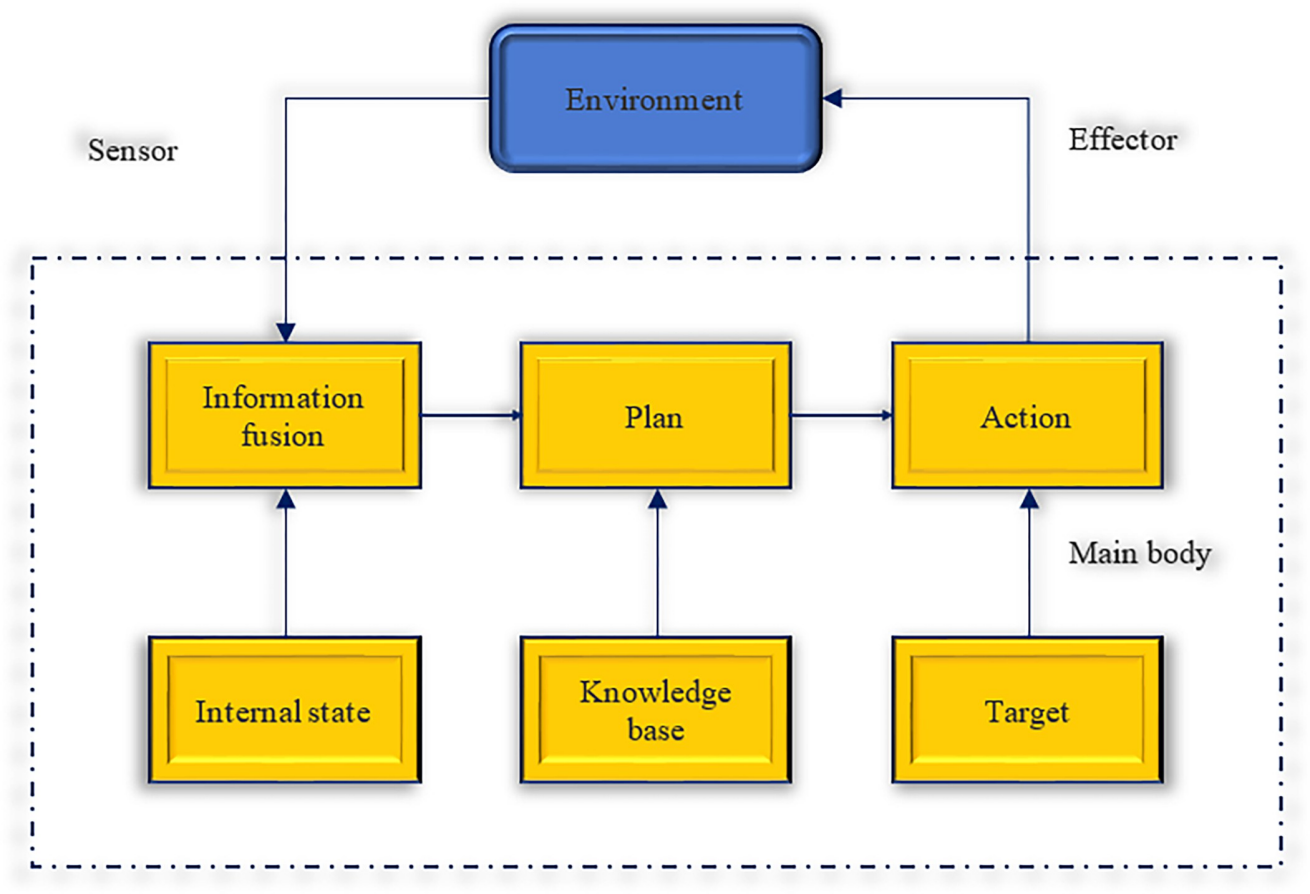
Inference architecture diagram.

The inference agent is developed from the thinking paradigm and has an internal, formalized, and reasoning model. It plans and negotiates to coordinate work with other agents [24]. Its structure is shown in Fig 1.

In Fig 1, the agent receives information about the external environment through sensors in an inference agent architecture. Information fusion is performed based on the internal state. Then, a description of the current state of modification is generated. Then, a plan is developed to form a series of actions with the support of the knowledge base. Finally, it acts on the environment through the effector.

**Fig 2:**
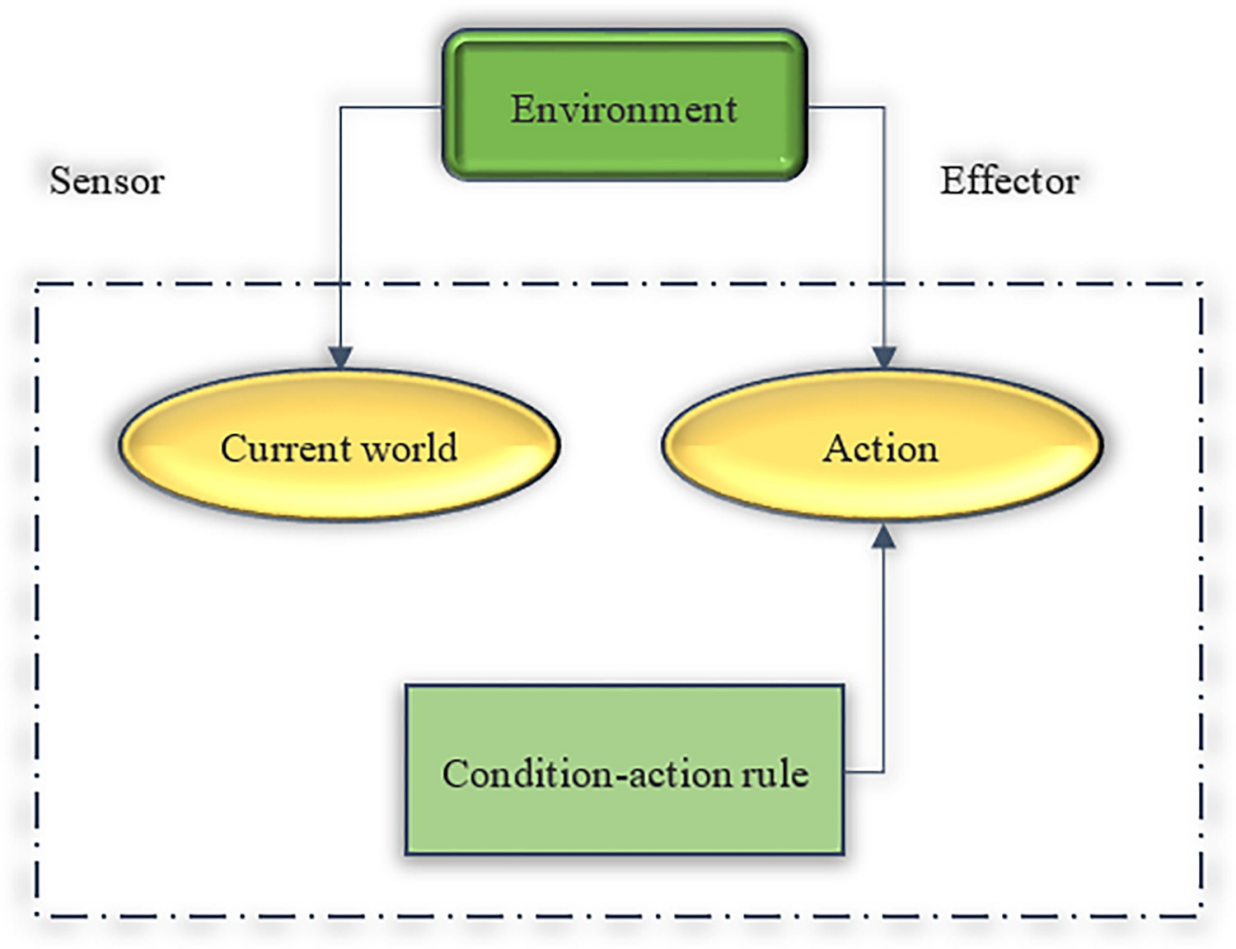
Reactive architecture diagram.

Reactive agent architectures do not have a formalized model internal to the environment. It simply reacts to the current state of the environment. Fig 2 displays its structure [25].

In Fig 2, conditional-action rules enable the subject to connect perception with action. The squares represent the current internal state of the subject during communication. The ellipse represents the background information used in the process. Reactive architectures currently dominate mainstream distributed systems.

(2) The operation process of the virtual enterprise

**Fig 3:**
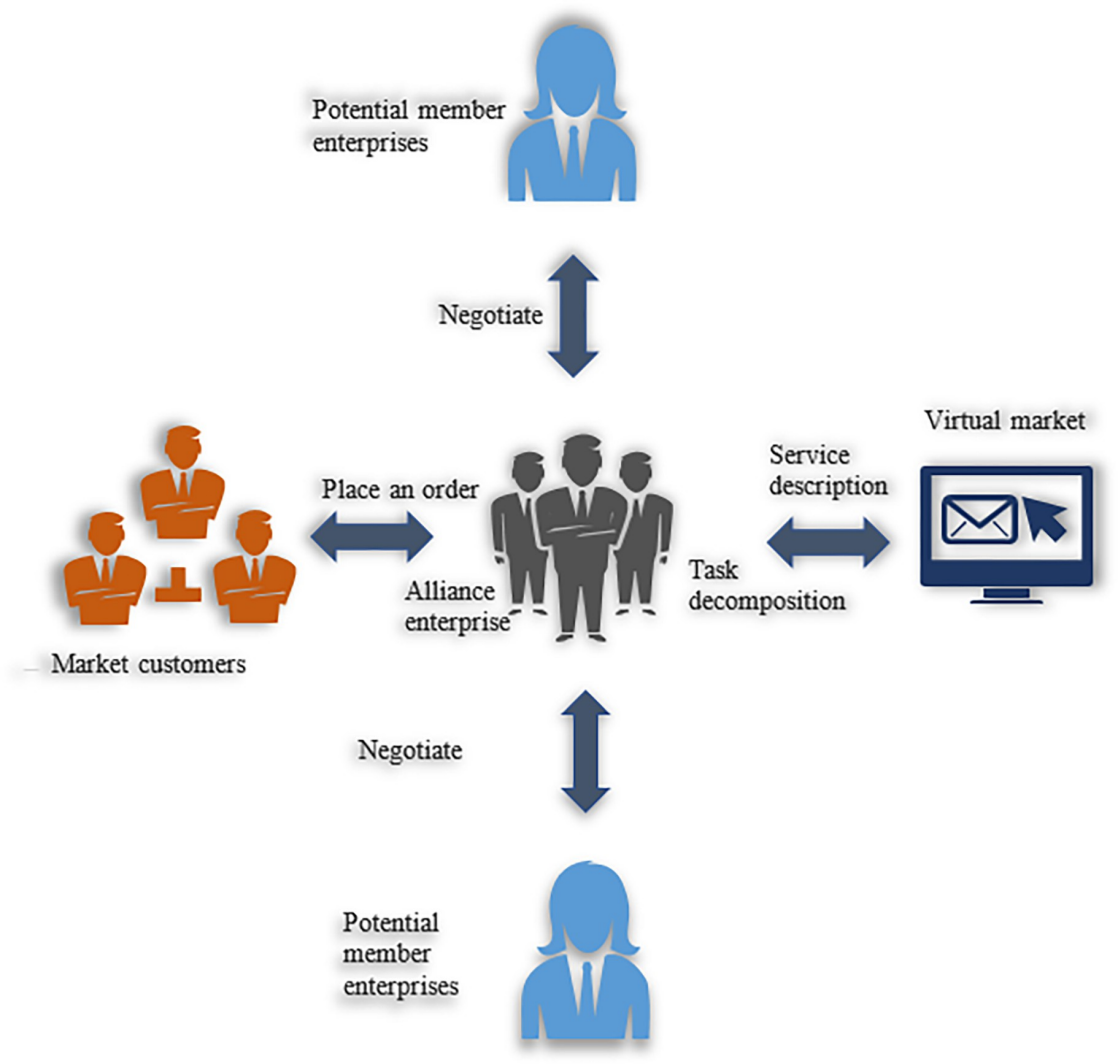
Virtual enterprise operating model.

Virtual enterprises are built according to market needs. The alliance enterprise plans the development and manufacturing scheme of the product in time according to the product structure and process flow to select partners to produce the products required by customers [26]. Thus, the operational process framework of product development for virtual enterprise is plotted in Fig 3.

Fig 3 signifies that when the alliance receives the project, it analyzes the requirements beyond its production capacity and consults the alliance from the virtual market. The alliance is responsible for managing the operation of the entire virtual enterprise to produce the potential member enterprises. Before the formation of virtual enterprises, establishing alliance status for the manufacturing industry with sophisticated equipment or a unit with extraordinary design capabilities depends on the product’s characteristics and the alliance’s business scope. At the end of the entire product lifecycle, ally status is canceled. The virtual marketplace is a third-party service provider that provides intermediary services for virtual enterprises’ dynamic formation and operation. The marketplace manages certain service types and provides shared domain ontology and service-matching mechanisms. Then, enterprise alliances can find suitable partners [27].

(3) Virtual enterprise negotiation communication model based on MAS

1) Model construction

**Fig 4:**
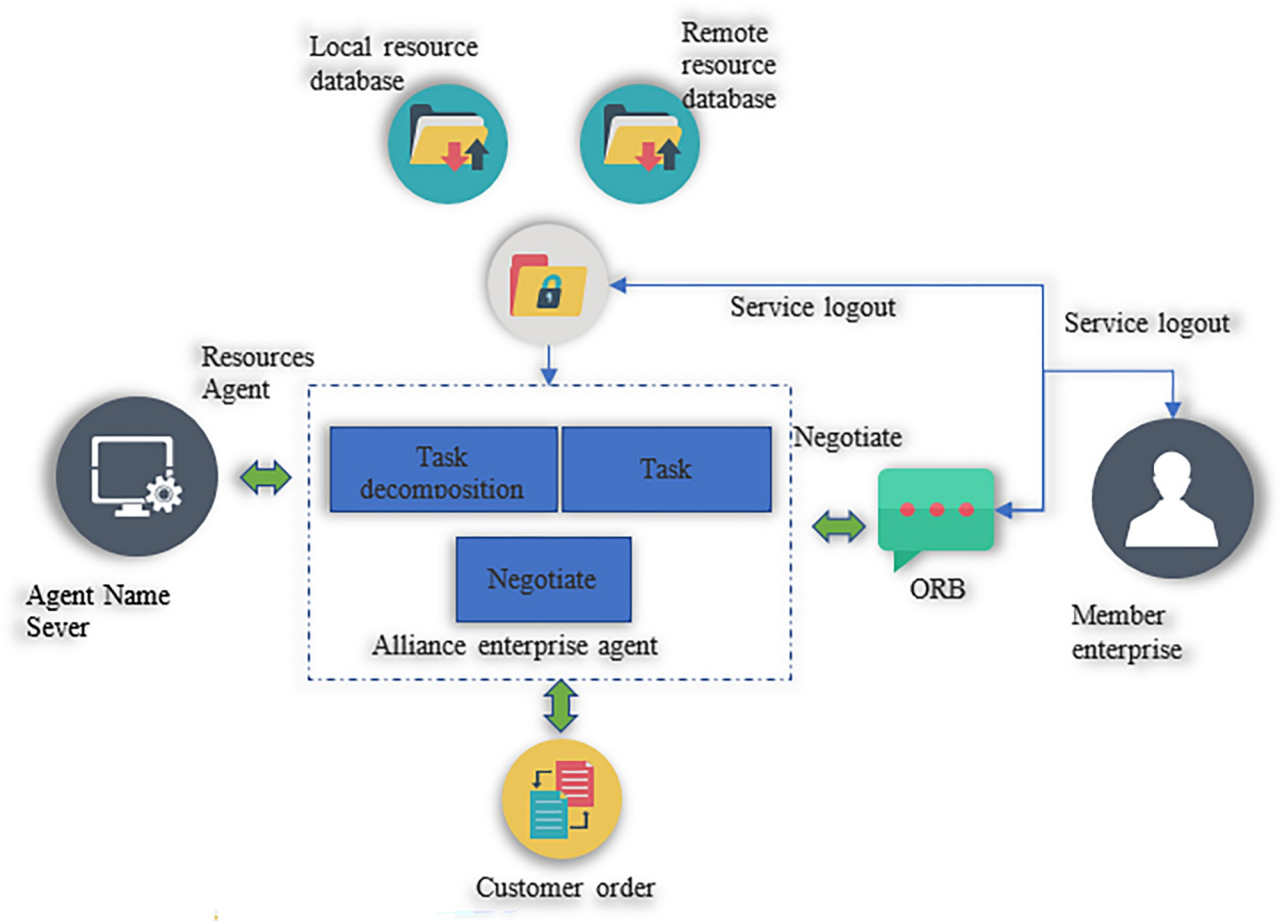
Virtual enterprise negotiation communication model.

According to the steps of modeling multi-agent technology, Fig 3 is broken down by function. The negotiation and communication model based on the virtual enterprise displayed in Fig 4 is constructed. The communication between agents and their structure is given. Furthermore, the alliance agent has three main sub-agents: the task agent, the task decomposition agent, and the negotiation agent.

In Fig 4, the task agent module is responsible for supervising and managing the whole process of task execution, including bidding and utility evaluation for each subtask. The task decomposition agent module accepts information for customer orders at the beginning. It preliminarily plans the entire project based on the tasks and content of order in the planning document. It also decomposes, receives, and processes task information. Different sub-tasks are formed to complete the initial planning. The resource agent module is mainly responsible for integrating local and off-site resources. The negotiation agent module is principally responsible for determining the negotiation strategy of the alliance enterprise and negotiating with the member enterprise. The member agent module means that each potential partner constitutes a member enterprise. It determines the negotiation strategy with the alliance enterprise and manages the tasks it has undertaken according to its own and external environment.

2) Design of Agent structure in virtual enterprise MAS model

**Fig 5:**
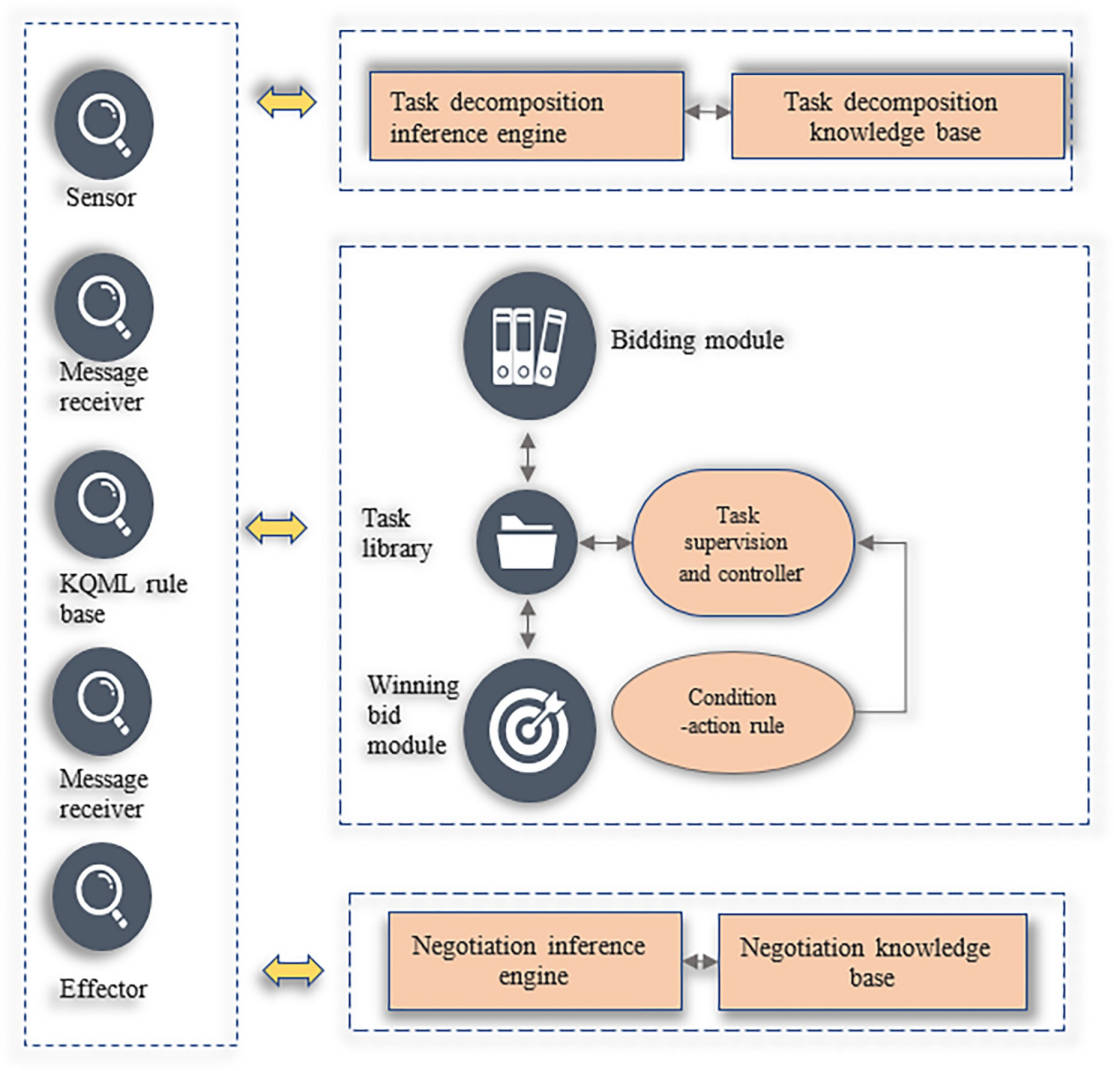
Structure diagram of the agent of the alliance enterprise.

Fig 5 exhibits the hybrid alliance enterprise agent structure for virtual enterprises.

In Fig 5, the task supervision and controller in the task agent are composed of a series of condition-action rules to improve the response of the alliance enterprise agent to the task, showing reactive characteristics. The bidding and winning modules in the task decomposition agent, negotiation agent, and task agent will respond intelligently according to changes in the internal and external environment, showing the reasoning characteristics. The role of the communication layer is to sense changes in the external environment through sensors and realize the effect on the environment through effectors. It is used to generate, send, and interpret messages.

3) Communication design between Agents in the virtual enterprise model

Regarding the communication between agents in the virtual enterprise model, the Knowledge Query and Manipulation Language (KQML) is selected as the technical support [28]. This language is currently the most widely used agent communication language based on the speech act theory. The purpose is to support the sharing and reusing of knowledge and information in a distributed, heterogeneous, dynamic, and many autonomous nodes (agents) environment. At present, it has become the de facto standard of agent communication language [29].

As a message-based communication protocol, KQML is also an independent information exchange and protocol language. KQML has three independent systems: independent network transmission mechanism, content language, and content entity. In addition, KQML is extensible. For systems in different application areas, it can define new behavioral words as long as these behavioral words still conform to the specification and have some practical function [30].

4) Negotiation characteristics of virtual enterprises

**Fig 6:**
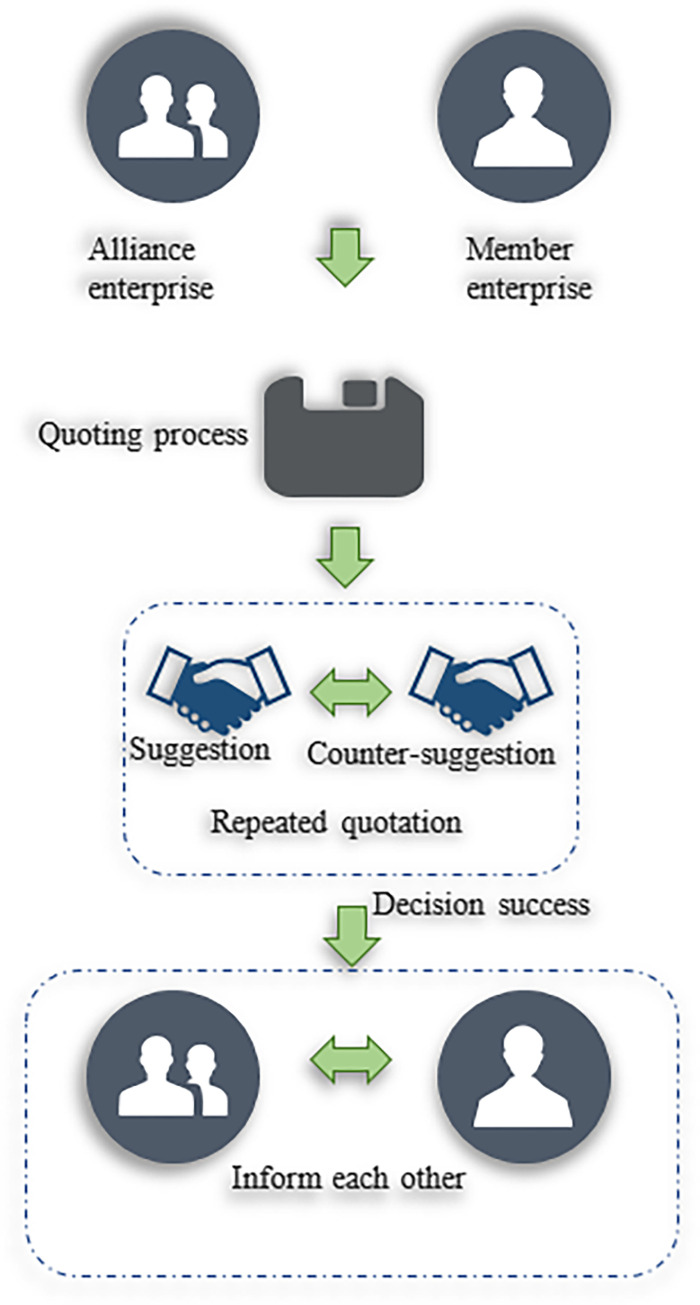
Negotiation agreement process for a virtual enterprise.

In a virtual enterprise environment based on a multi-agent, the agent represents the member companies of the virtual enterprise. The negotiation process agreement is portrayed in Fig 6.

Fig 6 shows the characteristics of negotiations between virtual enterprises. The agent is selfish but not wholly selfish. Negotiation objectives can include multiple objectives, such as price and time. The agent’s information is private. Agents have time constraints and resource constraints [31].

### Self-learning negotiation algorithm based on improved Bayesian

In the last section, virtual enterprise technology and MAS are used to complete the construction of the enterprise negotiation model. Then the specific algorithm adopted in the model will be designed. First, the theoretical framework of multi-topic negotiation for virtual enterprises is designed, and then the Bayesian algorithm is employed to implement the negotiation model further. In addition, the Bayesian algorithm will be improved to some extent to construct the enterprise negotiation model better.

(1) Design of the framework for multi-topic negotiation of virtual enterprise

The model of multi-topic negotiation for virtual enterprises can consist of a multivariate {*NA*, *NT*, *ACTION*, *X*, *V*(*X*), *Ω*, *T*, *a*(*T*), *U*}. *NA* is a collection of alliance and member enterprise agents in negotiation subtasks. *NT* is a sub-task for conducting negotiations. *X* means the topic vector for all topics covered in the talks. *V*(*X*) represents the agent’s multi-topic value vector. *Ω* means the value range of negotiation topic *X*. *T* expresses the negotiation time. *ACTION* refers to agents’ activities in a negotiation, including *Accept*, *Reject*, and *Counter Propose*. Therefore, Eq (1) can be obtained.


$${RESPONSE}_{b}\left(t,v_{h,t}^{x_{m}}(i)\right)=\left\{ \begin{array}{c} Reject,if\;t>t_{max}\\ Accept,if\;U_{v_{h,t}^{x_{m}}}^{b}(i)\geq U_{v_{b,t}^{x_{m}}}^{b}(i)\\ Counter\;Propose,\;otherwise \end{array}\right.$$ (1)

In Eq (1), *b* indicates the member enterprise agent. *h* represents the alliance agent. $v_{h,t}^{x_{m}}(i)$ indicates the proposal made by the alliance agent to the member enterprise agent on the topic *x*_*m*_ of the subtask *i* during the *t* round of negotiations. Similarly, $v_{b,t}^{x_{m}}(i)$ indicates a counter-proposal made by the member enterprise agent to the alliance agent on the topic *x*_*m*_ of subtask *i* during the *t* round of negotiations. $U_{v_{h,t}^{x_{m}}}^{b}(i)$ refers to the utility function of the member enterprise agent for the recommendation $v_{h,t}^{x_{m}}(i)$. *t*_*max*_ expresses the maximum number of negotiating rounds. It is the minimum number of negotiation rounds set by the alliance and member enterprises. The same is true for the agent’s *RESPONSE*.

Besides, *T*_*max*_ is set as the negotiation deadline $T_{max}^{h}.T_{max}^{h}$ is the deadline for alliance negotiation, as indicated in Eq (2):

$$T_{max}^{h}=u∙f(\frac{Num(b)}{\sum t_{h\to b}})$$ (2)

In Eq (2), $f(\frac{Num(b)}{\sum t_{h\to b}})$ represents *T*_*max*_ proportional to the number of agents that negotiate and inversely proportional to the time of negotiations. The function *f* needs to be determined according to the actual situation. *t*_*h*→*b*_ is the negotiation time set by the alliance with the member enterprise. *Num(b)* means the number of agents of the member enterprises in the collection participating in the bidding. *u* refers to the reasonable time the alliance believes in negotiating with a single agent. a(T) stands for the proposal parameter related to the agent risk trend in the negotiation. It is a parameter that changes over time *t*. Besides, $a^{x_{m}}(t)$ indicates the proposal parameter for the topic *x*_*m*_, and:

$$a^{x_{m}}(t)={\left(\frac{min(t,T_{max}^{h})}{T_{max}^{h}}\right)}^{\frac{1}{\beta }}$$ (3)

In Eq (3), *β* is the coefficient. When it takes different values, it corresponds to different negotiation strategies (conservative, intermediate, and risk-taking). *U* is the utility function. Regarding the topic *x*_*m*_, assuming that the agent of the alliance enterprise is a subtraction function, its utility function reads:

$$U_{h}^{m}=(1-\frac{x-{min}_{h}^{x_{m}}}{{max}_{h}^{x_{m}}-{min}_{h}^{x_{m}}})$$ (4)

If the member agent is an increasing function, its utility function is defined as:

$$U_{b}^{m}=(1-\frac{x-{min}_{b}^{x_{m}}}{{max}_{b}^{x_{m}}-{min}_{b}^{x_{m}}})$$ (5)

(2) Self-learning negotiation algorithm based on improved Bayesian

In virtual enterprise negotiation, enterprises choose the corresponding negotiation strategy according to their situations, such as unilateral concessions, competitive strategies, collaborative strategies, sabotage negotiations, and delay negotiations. During the negotiation process, the enterprise estimates the other party’s reserve price, thereby improving efficiency. Here, an improved Bayesian virtual enterprise self-learning negotiation algorithm is proposed. It adds to the traditional Bayesian negotiation algorithm the estimation of the number of negotiations to the extreme value of the negotiating counterparty. The model uses Bayesian learning to obtain the information of the negotiator from the negotiation information. Then, based on the information, a proposal for the next round of negotiations is given [32].

The Bayesian-based self-learning mechanism is discussed, taking the negotiation process of the alliance agent as an example. During the *t*th negotiation, the alliance agent will make a counter-proposal on the issue.


$$v_{h,t}^{x_{m}}(i)=\bar{{min}_{b}^{x_{m}}}+a^{x_{m}}(t)({max}_{h}^{x_{m}}-\bar{{min}_{b}^{x_{m}}})$$ (6)

In Eq (6), $a^{x_{m}}(t)$ (t) is incremental. Over time, the alliance agent will eventually propose the price of ${max}_{h}^{x_{m}}.\bar{{min}_{b}^{x_{m}}}$ is unknown to the alliance agent. However, it is assumed that some possible values $B_{q}^{b}$ are used to estimate $\bar{{min}_{b}^{x_{m}}}$. Besides, *q* = 1, 2,…, *Q*. $B_{q}^{b}$ obeys the probability distribution $P_{0}(B_{q}^{b})$. When the negotiations begin, the alliance will give an initial offer of ${min}_{h}^{x_{m}}$ for the topic.

Similarly, the proposal of the member enterprise agent is:

$$v_{b,t}^{x_{m}}(i)={min}_{b}^{x_{m}}+(1-a^{x_{m}}(t))(\bar{{max}_{h}^{x_{m}}}-{min}_{b}^{x_{m}})$$ (7)

In Eq (7), ${max}_{h}^{x_{m}}$ is unknown to the member agent. It is supposed that some possible values $B_{q}^{h}$ are used to estimate $\bar{{max}_{h}^{x_{m}}}$. Also, *q* = 1, 2,…, *Q*. $B_{q}^{h}$ obeys the probability distribution $P_{0}(B_{q}^{b})$ [33].

Then, during the *t*th negotiation, when the alliance agent begins to change belief after receiving the proposal, it can get the value of $P_{t}<B_{q}^{b}|v_{b,t}^{x_{m}}>$. $P_{t-1}(B_{q}^{b})$ is a known prior distribution. $P_{t}<v_{b,t}^{x_{m}}|B_{q}^{b}>$ is an unknown posterior distribution. After that, the alliance agent will continue to revise its beliefs as the number of negotiation rounds changes. When the number of negotiations reaches $T_{max}^{b}$, the member’s bid is its minimum acceptable value of ${min}_{b}^{x_{m}}$. According to the full probability equation, the alliance can estimate the reserve price of the member enterprise agent $\bar{{min}_{b}^{x_{m}}}$. Similarly, in the *t*th negotiation, when the member enterprise agent receives the proposal, it begins to change beliefs. At this point, the value of $P_{t}<B_{q}^{h}|v_{h,t}^{x_{m}}>$ can be acquired. After that, the member enterprise agent will continue to revise its beliefs as the number of negotiation rounds changes. When the number of negotiations reaches $T_{max}^{b}$, the bid of the alliance is the maximum acceptable value of ${max}_{h}^{x_{m}}$. Finally, according to the full probability equation, the member enterprise can estimate the top price of the alliance agent $\bar{{max}_{h}^{x_{m}}}$ [34].

Additionally, since many symbols are involved in this section, each symbol and its meaning are summarized as the results outlined in Table 1.

**Table 1:** Symbols and their meanings.

Symbols	Meanings
*NA*	The set of the alliance Agent and the member enterprise Agent in the negotiation subtask
*NT*	The subtask of conducting negotiations
*X*	The topic vector of all the topics involved in the negotiation
*V(X)*	Multi-topic value vector of Agent
*Ω*	Range of negotiation topic *X*
*T*	Negotiation time
*ACTION*	Action set of Agent in negotiation
*b*	Member enterprise
*h*	Alliance enterprise
*t*	Number of negotiating rounds
*i*	Subtask
*x*_*m*_	Topic
$v_{h,t}^{x_{m}}(i)$	During the negotiation of the *t* round, the offer made by the alliance agent to the member enterprise agent on topic *x*_*m*_ for sub-task *i*
$v_{b,t}^{x_{m}}(i)$	During the negotiation of the *t* round, the counteroffer made by the alliance agent to the member enterprise agent on topic *x*_*m*_ for sub-task *i*
$U_{v_{h,t}^{x_{m}}}^{b}(i)$	The utility function of member enterprise agent for recommendation $v_{h,t}^{x_{m}}(i)$
*t*_*max*_	The maximum number of rounds of negotiation
*t*_*min*_	The alliance and member enterprises set the minimum number of negotiation rounds
*T*_*max*_	Negotiation deadline
*t*_*h*→*b*_	The negotiating time set by the alliance with member enterprises
*Num*(*b*)	The number of bidding member enterprise agents in the set
*u*	The alliance considers a reasonable time to negotiate with a single Agent
*a*(*T)*	Quotation parameters
$a^{x_{m}}(t)$	Quotation parameters for topic *x*_*m*_
*β*	Coefficient

(3) Example design

For negotiations with subtask *i* on the *x*_*m*_ topic (price), the *Ω*_*m*_ of the alliance agent is [80,100]. The value of the β is 0.3. It is a risk-taking strategy with utility as a subtraction function. The *Ω*_*m*_ of the member enterprise agent is [90,110]. The value of the β is 2. It is a conservative strategy, and the utility is an increasing function. Alliance agent’s estimate $B_{q}^{b}$ of low agent price of member enterprises is [85,95]. Besides, $P_{0}(B_{q}^{b})$ = [0.8,0.2]. The estimate $B_{q}^{h}$ of the top price of the member enterprise agent for the alliance enterprise agent is [95,105], and $P_{0}(B_{q}^{h})$ = [0.2,0.8]. In addition, it is assumed that the $T_{max}^{h}$ of the alliance agent and the member enterprise agent = $T_{max}^{b}$ = 15.

(4) Algorithm implementation

**Fig 7:**
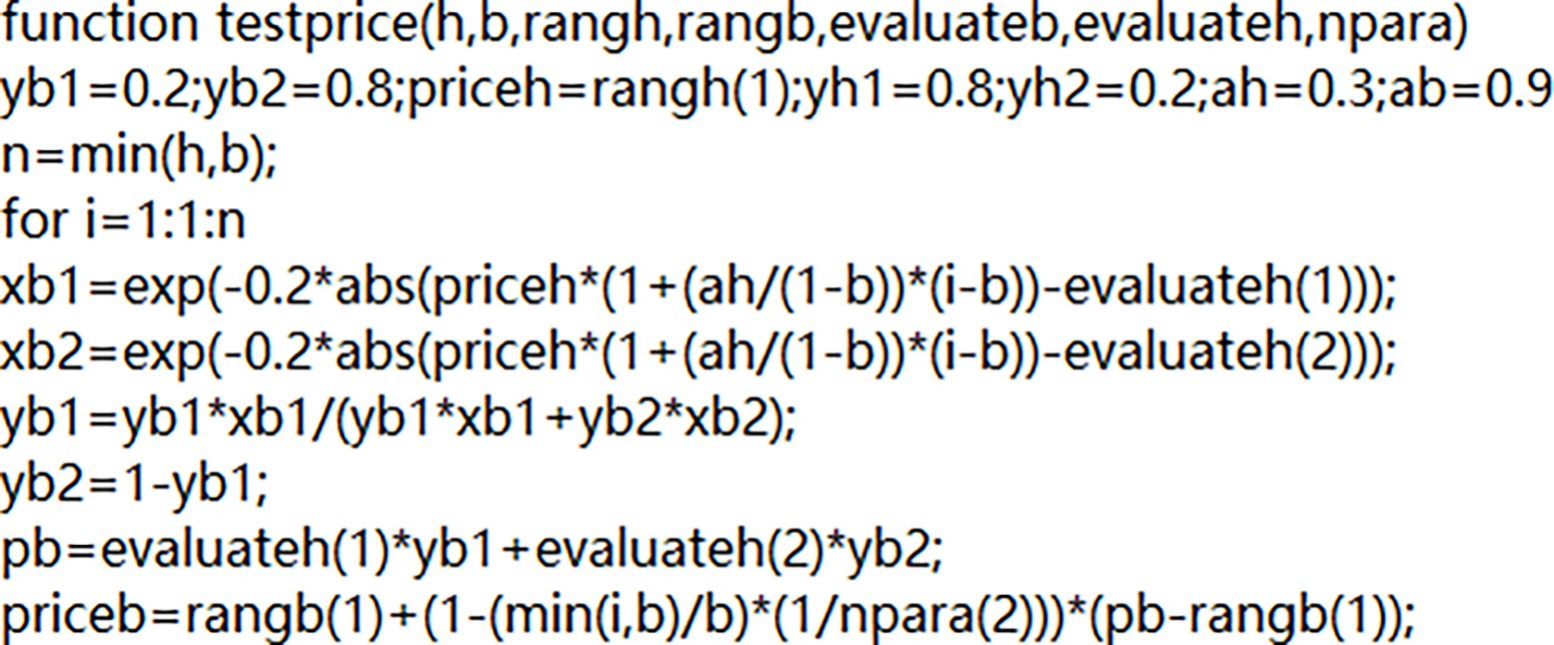
Partial code presentation of algorithm implementation.

MatLab platform is employed to implement the above-designed examples, and part of the code is presented in Fig 7.

Fig 7 describes according to the calculation examples designed above, the parameters can be run in the form of appropriate code to get the real-time decision effect of each example model. Furthermore, because the whole experiment is only uses the MatLab platform to complete the model, so only good performance of the computer can be, do not need to spend too much cost.

## Results

In the fields of distributed artificial intelligence (AI), information engineering, computer science, computer communication, and network, the study of a single agent and the MAS has always been a hot research topic. As one of the frontier directions, MAS is applied more and more widely. MAS can provide a more efficient means of solving complex scientific and engineering problems than traditional software systems. In recent years, the domestic and international research boom of the Internet of Things (IoT) is rising, and the IoT will be extensively used in all aspects of social life. By comparison, MAS and intelligent IoT have many similar things in common. That is, each terminal of the IoT can be regarded as a single agent. When they request communication and need a trusted connection, the networking problem of each terminal is similar to the alliance of a single agent. Their goal is the same: the agent’s communication process, access, processing, and interaction. For example, MAS’s trusted alliance technology can build multi-level security protection mechanisms in the context of the IoT. This mechanism can better protect network security, and this study is based on the research.

### The negotiation effect of the agent using different strategies

**Fig 8:**
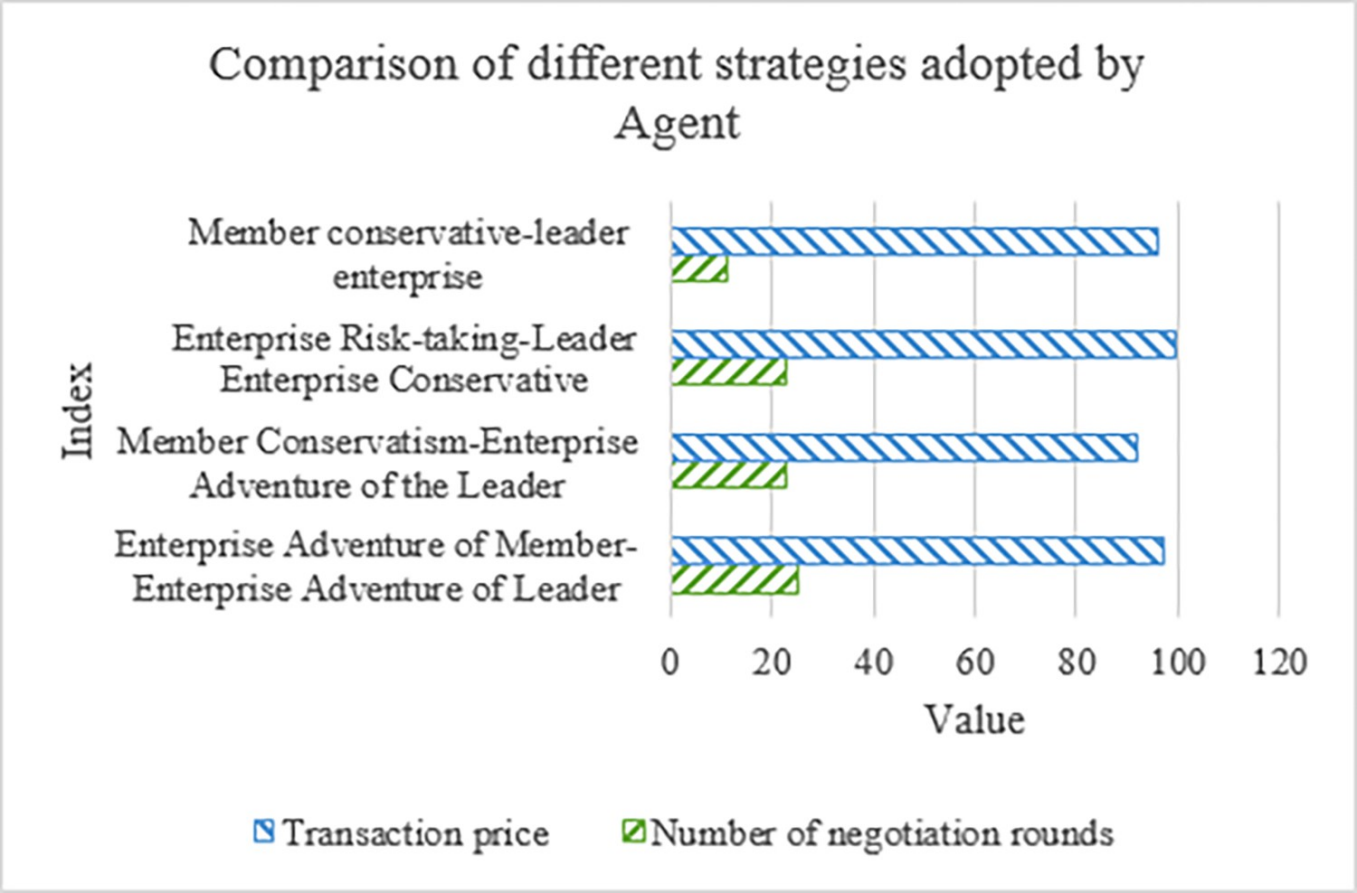
Comparison of different strategies used by agents.

The results of the negotiation using MatLab to obtain the different strategies adopted by the agent are revealed in Fig 8.

Fig 8 signifies the number of negotiation rounds and transaction prices using different strategies. In Fig 8, *T*_*max*_ = 30. It is found that when one side adopts a risk-taking strategy, the number of negotiation rounds between the two sides will increase dramatically. Additionally, it can be concluded from the transaction price that agents can obtain better joint effectiveness through learning. For example, the number of negotiation rounds of enterprise is significantly reduced after Agent learning.

### Negotiation effect under learned and unlearned agents

**Fig 9:**
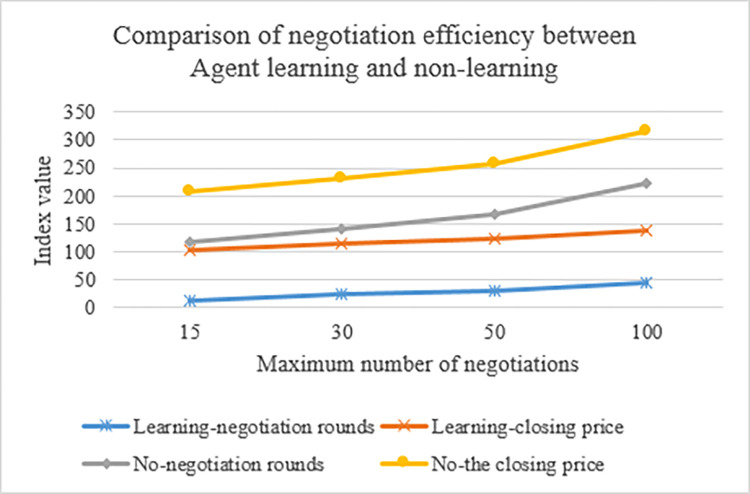
Negotiation efficiency compared to learned and unlearned agents.

The negotiation effect of learned and unlearned agents is compared to show that agents can improve negotiation efficiency through learning. Fig 9 denotes the results obtained after comparison.

Fig 9 demonstrates that when the agent does not learn, and the *T*_*max*_ takes 15, 30, 50, and 100, the number of negotiation rounds is 13, 26, 43, and 85, respectively. When the agent learns, and *T*_*max*_ takes the same value, the number of rounds of negotiation is 12, 23, 29, and 43, respectively. The above data indicate that negotiations’ efficiency increases as the number of negotiation rounds rises. The efficiency of agent negotiation after learning is significantly better than that of unlearned. From the transaction price, it is concluded that an excellent joint effect can be obtained through learning.

### A comparison of agent-improved Bayesian and traditional Bayesian negotiation efficiency

**Fig 10:**
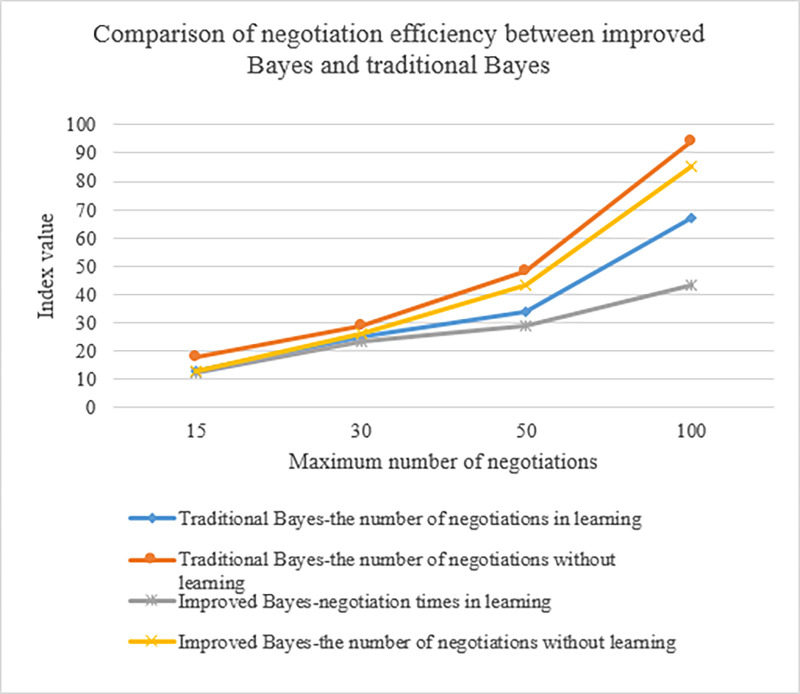
Comparison of negotiation efficiency between improved Bayesian and traditional Bayesian.

The negotiation effect of the improved and the traditional Bayesian algorithms is also compared to show that the negotiation effect based on the improved Bayesian algorithm can be promoted. Fig 10 reveals the results.

In Fig 10, for the improved Bayesian algorithm, when the agent is learning or not learning, its negotiation rounds are better than the traditional Bayesian algorithm. It implies that the improved Bayesian algorithm can improve the efficiency of negotiations by reducing the number of negotiation rounds.

### Comparison of the effect of the proposed model with other models

To further verify the effectiveness of the proposed Agent-based negotiation model, this model is compared with the PRAM negotiation model (making a Plan, establishing a Relationship, reaching an Agreement, fulfilling the agreement, and Maintenance of relationship). The comparison results are suggested in Table 2.

**Table 2:** Comparison between the proposed model and the PRAM negotiation model.

Evaluation index	The proposed model	PRAM model
Selfishness	Semi-selfish	Semi-selfish
Number of negotiations	One-to-many	Many-to-many
Negotiation attribute	Many questions	Single question
Private information	Secrecy	Open
Restrictions	Clear	Clear
Mobility	No	No
Priority of negotiation issues	Nothing	Nothing
Negotiation results	Feasible	Feasible

Table 2 outlines that the Agent constructed here is semi-selfish. Its information is private, and its goal is to maximize its interests, which reflects its selfish side. However, to improve the efficiency of the overall negotiation, the Agent also estimates the negotiation value, which demonstrates the aspect of cooperation. Negotiation is multi-objective, such as price, time, punishment, etc., which accords with the negotiation characteristics of virtual enterprises.

## Discussion

Currently, there are still some problems in the construction of the communication management system of enterprise negotiation, such as low efficiency of enterprise negotiation and easy to make mistakes in enterprise information management. On the basis of previous scholars’ research, this study employs the virtual enterprise theory and Agent technology to design the enterprise negotiation model. On the one hand, the core parameters contained in the model are mainly the quotation $v_{b,t}^{x_{m}}(i)$, and the number of negotiations *t*. Then, according to the functional relationship between the quotation and the number of negotiations, the optimal negotiation result is achieved. On the other hand, this model-based example is realized in the MatLab platform based on the Windows environment. Because of MatLab’s self-built function library, the model has the advantages of low implementation cost, simple and easy operation process, intelligent negotiation process, and so on. This study aims to improve the efficiency of enterprise negotiation and realize the efficient management of enterprise communication.

## Conclusions

In previous studies on the enterprise negotiation system, most discussed and studied this system based on a theoretical model or evaluation system, such as a dynamic alliance process model and a virtual enterprise organization design model. However, such models are not implemented by technical means. According to this, on the basis of previous studies of scholars, this study takes virtual enterprises as the research object and adopts Agent technology in the IoT to model the enterprise decision-making process. Then the traditional Bayesian negotiation algorithm is improved and applied to the negotiation process of the virtual enterprise, and an example is set to verify the effect of the algorithm. The results manifest that the joint effect can be obtained when both parties adopt a conservative strategy in negotiation. Moreover, the negotiation efficiency of the learned agent is significantly better than that of the unlearned agent. Beyond that, in the Agent-based enterprise negotiation model, the negotiation is multi-objective, which accords with the negotiation characteristics of a virtual enterprise. The disadvantage is that only the enterprise negotiation mechanism’s theoretical design has yet to be applied. The follow-up research will give the design process of virtual enterprise optimization application software based on the proposed negotiation decision-making mechanism. This study aims to use the improved Bayesian negotiation algorithm for the multi-topic negotiation process of virtual enterprises, which is significant for guiding enterprise practice.

## Supporting information

S1 Data (ZIP)
