# Supplementary material for: Enterprise negotiation and communication management system under the guidance of the Internet of Things
Source: PLoS One. 2023 Apr 25;18(4):e0284891. doi: 10.1371/journal.pone.0284891 (PMC10129010; doi:10.1371/journal.pone.0284891)
Supplement: S1 Data — (ZIP) [file pone.0284891.s001.zip › data/Figure 1.pptx]

## Slide 1
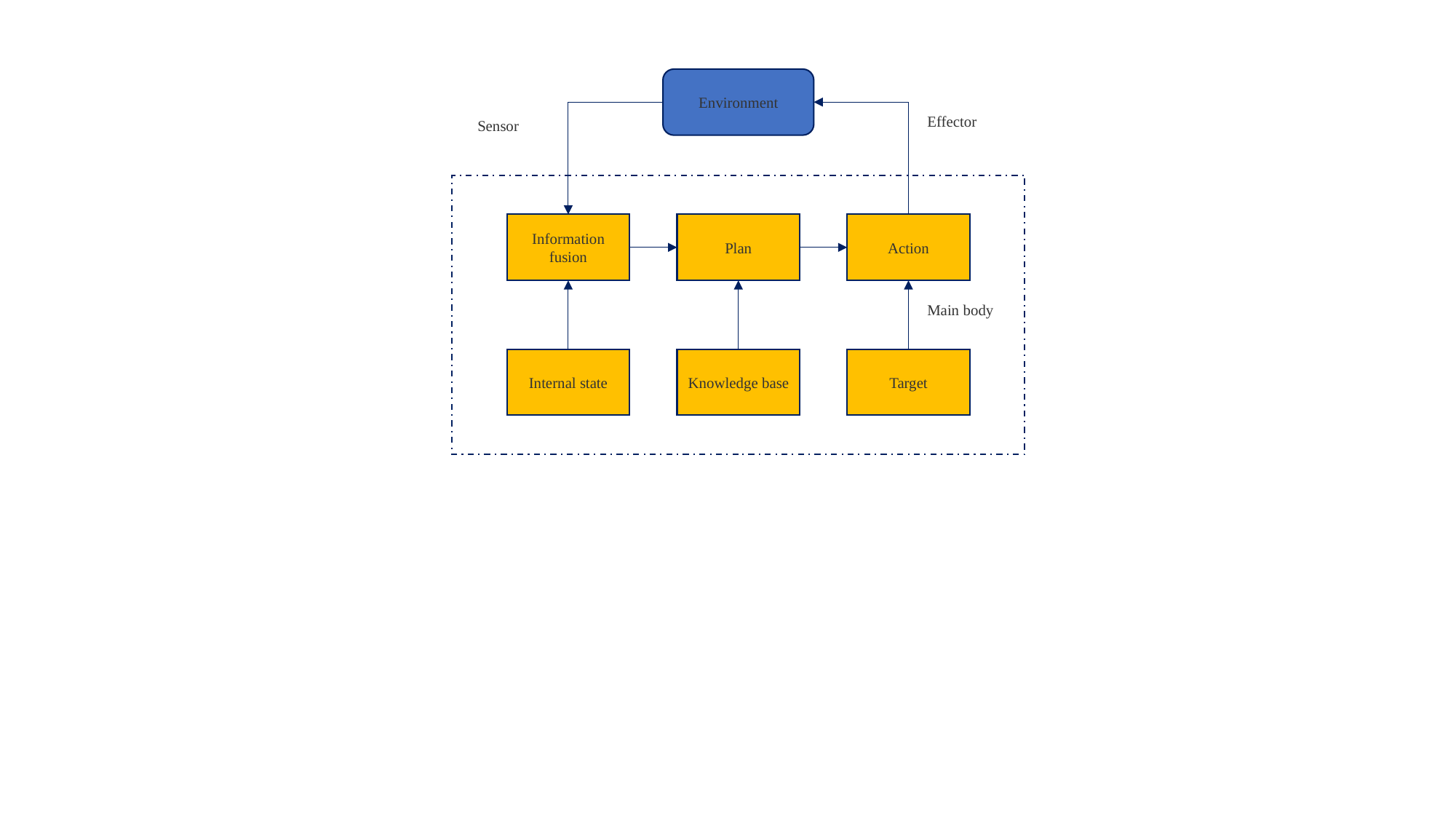

Environment
Effector
Sensor
Information fusion
Plan
Action
Main body
Internal state
Knowledge base
Target
