# Supplementary material for: Enterprise negotiation and communication management system under the guidance of the Internet of Things
Source: PLoS One. 2023 Apr 25;18(4):e0284891. doi: 10.1371/journal.pone.0284891 (PMC10129010; doi:10.1371/journal.pone.0284891)
Supplement: S1 Data — (ZIP) [file pone.0284891.s001.zip › data/Figure 3.pptx]

## Slide 1
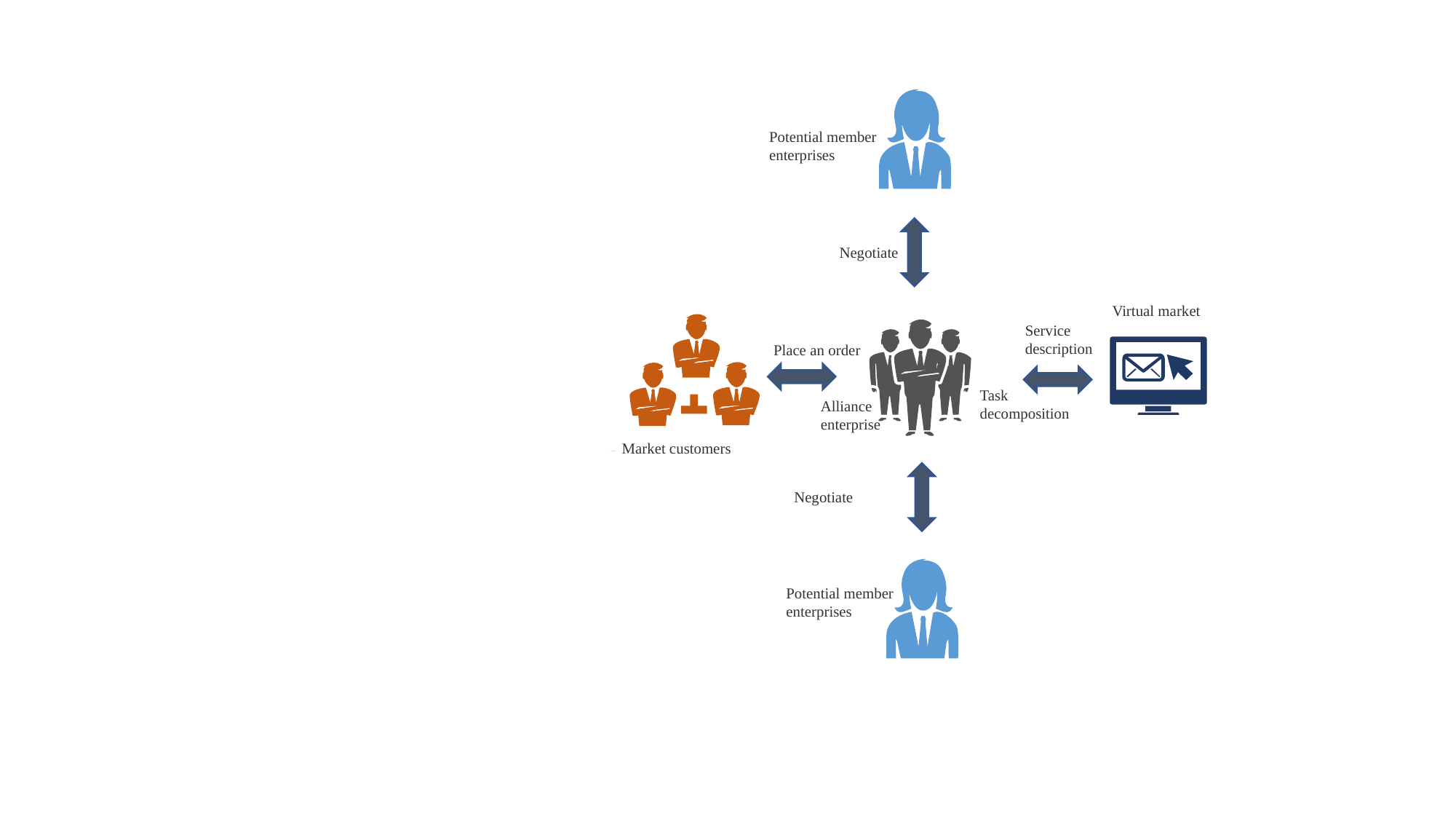

Potential member enterprises
Negotiate
Virtual market
Service description
Place an order
Task decomposition
Alliance enterprise
Market customers
Negotiate
Potential member enterprises
