# Supplementary material for: Enterprise negotiation and communication management system under the guidance of the Internet of Things
Source: PLoS One. 2023 Apr 25;18(4):e0284891. doi: 10.1371/journal.pone.0284891 (PMC10129010; doi:10.1371/journal.pone.0284891)
Supplement: S1 Data — (ZIP) [file pone.0284891.s001.zip › data/Figure 4.pptx]

## Slide 1
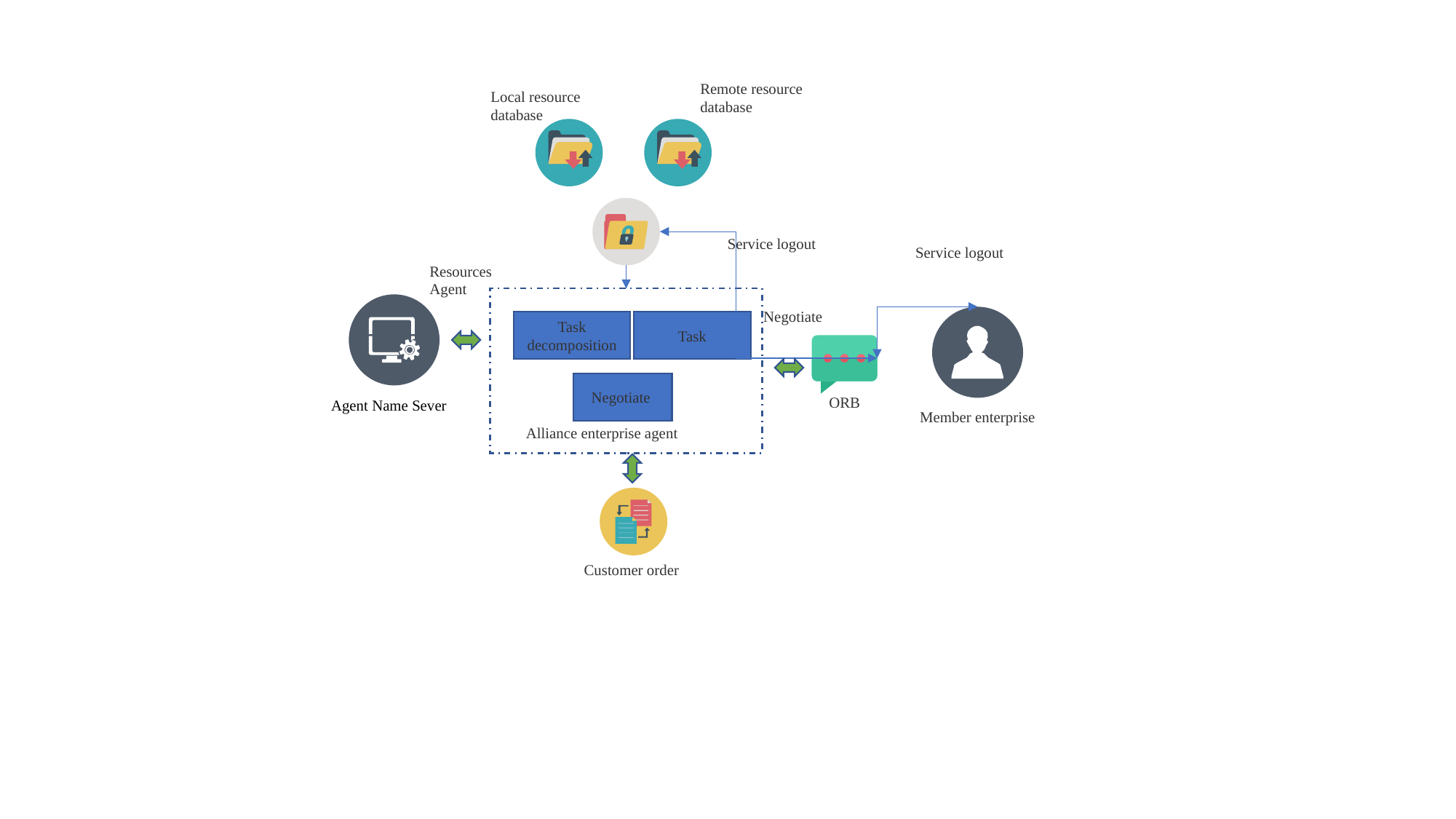

Remote resource database
Local resource database
Service logout
Service logout
Resources Agent
Negotiate
Task
Task decomposition
Negotiate
ORB
Agent Name Sever
Member enterprise
Alliance enterprise agent
Customer order
