# Supplementary material for: Enterprise negotiation and communication management system under the guidance of the Internet of Things
Source: PLoS One. 2023 Apr 25;18(4):e0284891. doi: 10.1371/journal.pone.0284891 (PMC10129010; doi:10.1371/journal.pone.0284891)
Supplement: S1 Data — (ZIP) [file pone.0284891.s001.zip › data/Figure 5.pptx]

## Slide 1
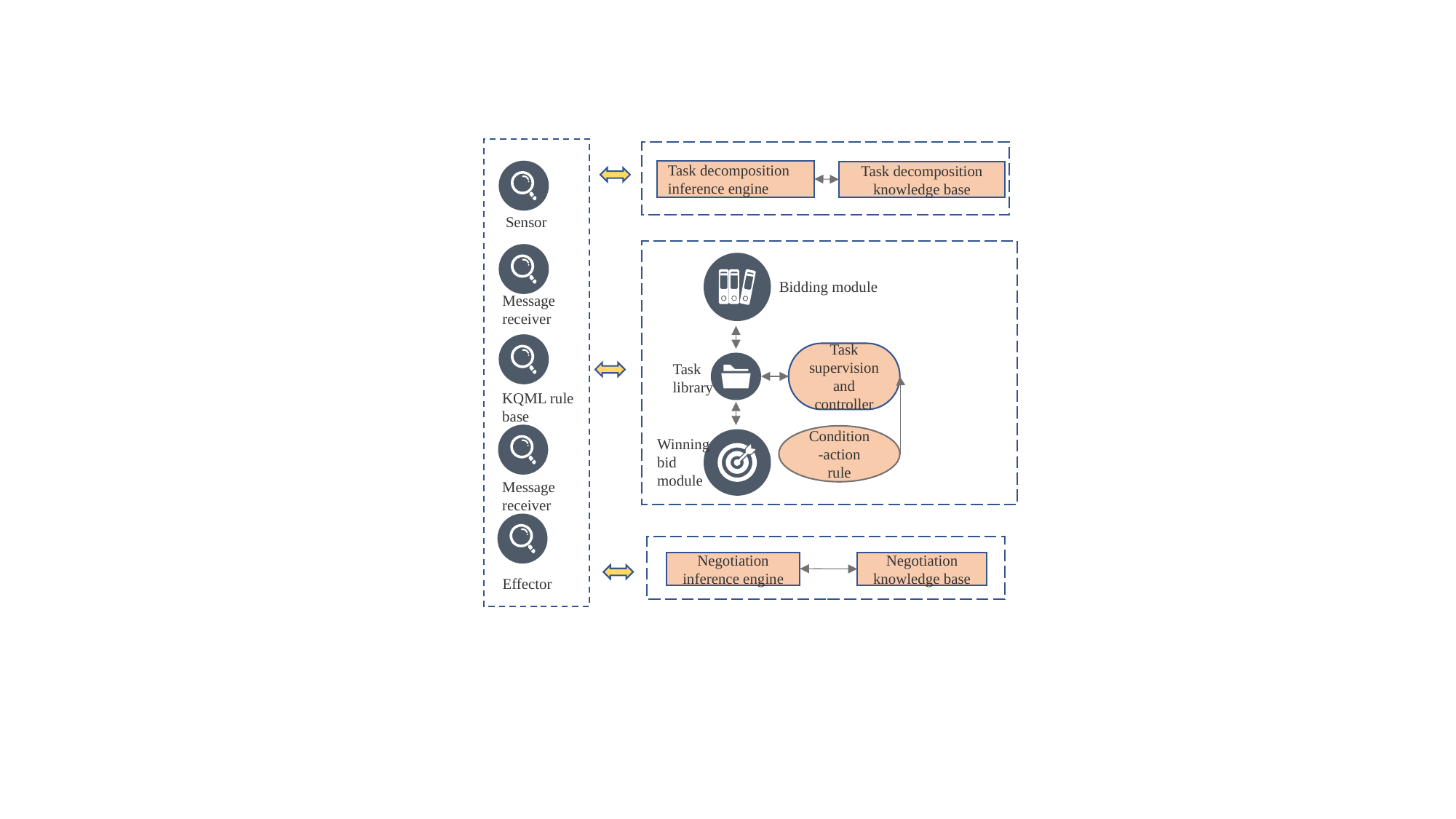

Task decomposition inference engine
Task decomposition knowledge base
Sensor
Bidding module
Message receiver
Task supervision and controller
Task library
KQML rule base
Condition-action rule
Winning bid module
Message receiver
Negotiation inference engine
Negotiation knowledge base
Effector
